# Use of Opium in Labour-Pains

**Published:** 1813-10

**Authors:** J. Collins

**Affiliations:** Surgeon. Coventry


### *To the Editors of the Medical and Physical Journal.*

GENTLEMEN,

IN the Medical and Physical Journal for July last, you have given us a very interesting communication from Dr. Spark, of Ipswich, upon the advantages derived from very large doses of opium, in what Dr. S. terms spasm of the uterus; but which might, I conceive, with more propriety, be called Rigidity of the transverse fibres of the Uterus. It is common with accoucheurs to have recourse to opium for relief, in cases of this nature; but I am not aware that it has ever been given to the extent Dr. S. advises; and having lately met with two cases, in which the result of the practice he recommends has been most favorable, I beg leave, through the medium of your valuable publication, to present them to my medical brethren, together with a few observations upon the nature of such cases.

Case 1st.—About six o'clock on Sunday evening, July 19th, I was called to Mrs. K. of this city, aged 19, in labor of her first child. Her pains had commenced the preceding night, and continued almost incessantly during the day. Upon examination, I found the head low down in the pelvis, the os uteri not more dilated than the circumference of a shilling, and that the pains did not appear to produce the least effect upon it. She had taken but little nourishment, had no sleep, her pulse was very feeble, and she was much exhausted. I immediately gave her fifty drops of laudanum, and directed that small quantities of nourishing food should be frequently administered. At ten o'clock I called upon my patient again, and was informed by her attendants that she had slept a short time after the exhibition of the draught, and had taken freely of mutton broth; yet her strength did not appear recruited, nor her pulse improved. Her pains were still, what are termed grinding; and the dilatation of the os uteri had not made the least progress. Encouraged by Dr. Spark's practice, I repeated the laudanum in the same quantity; and directed them to send for me when they should think it necessary. About one o'clock I was again called, but the child was born before I could reach the house. The nurse informed me that Mrs. K. slept about an hour after she had taken the second draught, that she awoke much refreshed, and entirely free from pain, which had not been the case during the whole of the preceding day and night; and that she continued so for at least half an hour afterwards. The nurse, as well as the friends of the patient, assured me that she had not more than four or five five strong pains before the child was born. The placenta came away in about ten minutes, and no hemorrhage followed.

Case 2d.—At ten o'clock on the evening of the 8th of August, I was sent for to Mrs. H. of this city, aged 31, in labor of her first child. The head was low in the pelvis, and the os uteri but little dilated. The pains had been frequent since the morning of the 7th. In this case the patient's strength was good. Finding, upon examination during a pain, that little or no impression was made upon the os uteri, I gave her fifty drops of tincture of opium, and left her. Between twelve and one I was again called, but found not the least difference in the nature of the pains, in their continuance or effect; upon ascertaining which I gave her forty drops more of the tincture, and desired I might be called when it should seem necessary. At half after three they sent for me again, and, as I arrived at the bed-side of my patient, the head was passing the external parts. The placenta was set at liberty by the same pain which expelled the body of the child, and only required to be removed from the vagina. She informed me that she had no sleep after the second draught, but lay for some time very easy and comfortable; and when her pains returned, they increased very rapidly in strength, and were (to use her own words) in her belly, and not in her back as before. No hemorrhage ensued.

I was exceedingly gratified, in both of these cases, by the effects resulting from the employment of laudanum, the use of which proved highly beneficial, and was in neither instance succeeded by any unpleasant consequences. On examination (in the first case) even at ten o'clock, not the least relaxation either of the os uteri or external parts was perceptible: on the contrary, the latter were unusually contracted, firm, and rigid, and I could not make my examination without occasioning much pain.

Considering, therefore, all the circumstances, together with the size of the child, which I should have said was large, I have no hesitation in believing, judging from similar cases, that, had not a quantity of opium sufficient to have produced a decided effect upon the system been given, the passage of the head through the external parts, even after the dilatation of the os uteri, would have been slow and tedious, if effected without the assistance of the forceps.

The result of the use of opium in the second case was equally striking, and I imagine no one will differ from me in thinking, that, had it not been administered, the dilatation of the os uteri, which made such little progress in so many hours, hours, and the passage of the first child of a woman 31 years of age through the external parts, would not have been accomplished in a space of time little exceeding two hours.

Dr. Spark assures us that in this practice "the spirits of the woman are exhilarated, the uterus performs its functions with vigor, it gives way rapidly to the pressure of the child, the placenta never adheres, hemorrhages never follow, the uterus retains nothing, of course the patient is not afflicted with after-pains, and she recovers her health and strength more quickly than those who need not the aid of opium." Perhaps it may be thought that the doctor must have been too sanguine in the above passage: however this may be, the cases I have related are certainly corroborative of his statement; and we are naturally led to enquire how and in what manner these effects are produced. The most rational mode of ascertaining this appears to be an examination of the structure of the uterus itself, (as far as is necessary for our purpose,) and a consideration of the means by which a natural delivery is effected. The muscular fibres of the uterus are found to run in a transverse and a longitudinal direction. The action of the transverse fibres will evidently be that of closing the os uteri, and of flattening the body of this viscus; the action of the longitudinal will approximate the fundus and os uteri, and dilate the latter. The mutual and co-equal action of both will have the effect of embracing and retaining the contents of the uterus. Hence then it will appear that the expulsion of the child must be accomplished by the longitudinal fibres, (assisted by the abdominal muscles,) with the consent or relaxation of the transverse. The greater the degree of rigidity or want of relaxation which may be present in the transverse fibres, the longer will the grinding pains continue, which are nothing more than the contraction of the longitudinal opposed to the transverse in the dilatation of the os uteri.

If then this view of the subject be correct, we at once perceive in what manner the opium produces its good effect. That it causes relaxation in the transverse, and allows the longitudinal fibres to act without opposition; or to be more explicit, the contractions of the whole of the muscular fibres of the uterus being removed, we can easily imagine the longitudinal will be roused from their state of relaxation (by that power which called them into action) before the opium has ceased to exercise its uninterrupted influence over the transverse.

Bleeding has been recommended to facilitate labour, which it no doubt would do by producing the same effect as opium;

 but but at an unwarrantable expence to the constitution, except in cases of absolute necessity.

Dr. Spark observes that the tincture of opium has not the effect in any dose. This I could not conceive possible if retained in the stomach; and having the tincture at hand in the first case, when some time must have been lost in procuring the crude opium, I ventured to give it, and its use was followed by the desired effect. The same convenience, and the result of the first case, induced me to have recourse to it in the second.

If it were possible to ascertain the precise quantity of laudanum that would be necessary to take off the action of the uterus, it would be advisable not to give one drop more than that quantity, because, though we are desirous of producing relaxation of the transverse fibres, we should wish to interfere as little as possible with the longitudinal. Might it not, therefore, be advisable to give the requisite quantity of opium in divided doses at short intervals, rather than in six, eight, or ten grains at once, according to the degree of spasm, as Dr. S. recommends, which must be a fallacious mode of ascertaining the necessary dose?

I am, Gentlemen,

*Coventry*, Your's, &c.

*August* 14, 1813. J. COLLINS, Surgeon.

P. S.—Since writing the above, I have met with a case, which is by no means uncommon, but which might, I think, with more propriety, be referred to spasm. The woman had suffered a month from very frequent pains in her back; her nights had consequently been sleepless. She had frequently been afflicted with diarrhœa, accompanied by tenesmus, which, however, did not appear to affect the general health in any other way than by inducing debility. In this case, no symptom of labour excepting pain was present. I did not, therefore, think it prudent to give opium to that extent I should have done had she been actually in labour, but directed her to take a pill containing two grains of this substance every night at bed-time. In the morning, after taking the first dose, I found that she had passed a good night, had been, and still remained, free from pain. In the course of the day symptoms of labour came on, and she was delivered before nine in the evening.

Sept. 6th.—The ideas which I have ventured to suggest of the modus operandi of opium in parturition, induced me to expect that the grinding pains of labour might, in all cases, be greatly alleviated, if not entirely removed, by its proper proper use; and dispelled the fears I had formerly entertained of hemorrhage being a probable consequence of this practice. Had I not already trespassed very long upon your time and attention, I would have related several cases of natural labour in which I have given it with complete success. J. C.

